# Nanostructures for sensors, electronics, energy and environment II

**DOI:** 10.3762/bjnano.6.197

**Published:** 2015-09-23

**Authors:** Nunzio Motta

**Affiliations:** 1 School of Chemistry, Physics and Mechanical Engineering and Institute for Future Environments, Queensland University of Technology, 2 George St., Brisbane 4001, Australia

**Keywords:** electronics, energy, environment, nanostructures, sensors

This Thematic Series, “Nanostructures for sensors, electronics, energy and environment II”, is a continuation of the series released three years ago and again presents articles in this highly dynamic field. The fields of nanoscale science and technology are rapidly emerging, with a focus on the design, fabrication, and characterization of functional objects. The existing energy crisis could be mediated not only by new and more efficient methods of collecting sunlight, but also by saving resources by applying developments in storage, electronics and sensors.

Conventional energy sources are limited and most of them generate greenhouse gases that pollute the environment. Photovoltaic technology is a potentially viable solution to produce clean energy; however, its production costs are still too high due to the materials and process techniques involved. Moreover, because the sun is an intermittent energy source, the further development of energy storage systems is necessary in order to allow photovoltaic-based power generation to be independent from the grid.

Carbon, one of the most abundant materials found on earth, is the key atomic species in the compounds responsible for greenhouse gas emission and pollution. However, it can also be used to offset these effects, acting as a valuable material for energy generation, storage, carbon sequestration [1] and sensing [2–3]. Carbon can be employed in one or more of its allotrope forms (e.g., graphene, carbon nanotubes, fullerene) in devices such as organic and inorganic solar cells and supercapacitors [4]. These devices can be produced in large quantities with inexpensive synthesis and process methods based on printing and roll-to-roll techniques, establishing the basis of a new green technology.

Currently, most of the research effort in the field is focused on the synthesis of large quantities of high quality carbon nanomaterials in order to use them for industrial scale production of energy generation and storage devices. However, other interesting advances are appearing and are covered in this series.

Graphene and graphene oxide exhibit interesting properties that can be exploited in room temperature gas sensing devices. The plasmonic effect, generated by the inclusion of metallic nanoparticles, can be used to overcome certain limitations of the carbon materials, especially in organic solar cells [5].

The optical properties of nanomaterials can also be exploited to produce new, powerful devices such as nanolasers, light emitting devices [6] and optical nanosensors.

Nanomaterials continue to intrigue researchers with new properties discovered every year in such low dimensional structures, generating an incredible field of basic and applied research with the expectation of achieving a better, cleaner and more sustainable world.

Nunzio Motta

Brisbane, September 2015
